# 2693. Epidemiology of Infections with Multidrug-Resistant Organisms in Patients with Left Ventricular Assist Devices (LVADs)

**DOI:** 10.1093/ofid/ofad500.2304

**Published:** 2023-11-27

**Authors:** Dierdre B Axell-House, Radhikesh Ranadive, Tariq Nisar, Mahwash Kassi, Ashrith Guha, Cesar A Arias

**Affiliations:** Houston Methodist Research Institute, Houston, Texas; Houston Methodist, Houston, Texas; Houston Methodist Research Institute, Houston, Texas; Houston Methodist Hospital, Advanced Heart Failure and Transplant, Methodist DeBakey Cardiology Associates, Houston, Texas; Houston Methodist Hospital, Advanced Heart Failure and Transplant, Methodist DeBakey Cardiology Associates, Houston, Texas; Houston Methodist and Weill Cornell Medical College, Houston, TX

## Abstract

**Background:**

Left ventricular assist devices (LVADs) constitute one of the limited options available to patients with refractory heart failure. Infection is the most common adverse event after LVAD placement, and difficult to eradicate when attributable to the LVAD itself. LVAD-attributable infections (LVADI) thus lead to significant antibiotic exposure and higher risk for antibiotic resistance. However, the epidemiology of multidrug-resistant organisms (MDROs) causing LVADI has not been described.

**Methods:**

We conducted a retrospective cohort study of patients (pts) ≥18 years old with LVADs implanted from 2016 to 2022 at our institution. Infections were defined as LVADI if i) there was a positive culture from LVAD components, with or without concomitant blood cultures, or ii) there were positive blood cultures with radiologic evidence of LVADI. MDROs were defined according to the Centers for Disease Control (CDC) criteria. Primary outcome was mortality, and multivariate logistic models were built and adjusted for patient demographics and Charlson’s Comorbidity Index.

**Results:**

A total of 252 pts were implanted with new LVADs, with 104 (41.2%) pts developing LVADI. Of the 52 episodes of bloodstream infection (BSI) in 41 pts, 36.5% were caused by MDRO, with Methicillin-Resistant *Staphylococcus aureus* (MRSA) and MDR *Pseudomonas aeruginosa* most frequent. There were an additional 64 pts with 99 infections with positive LVAD component cultures, with a total of 115 pathogens isolated. Of these, 20% were MDRO, with MRSA and MDR Enterobacterales most frequent. Multivariate logistic models showed that pts with LVADI did not have higher odds of mortality (OR =1.61; 95% CI [0.92 – 2.81]; p=0.1), however pts with MDRO LVADI had a greater than 3 times odds of mortality (OR = 3.34; 95%CI [1.68 – 6.64]; p< 0.001) compared to pts without MDRO LVADI.

**Conclusion:**

Our review provides initial insight as to the burden of antibiotic resistance among bacteria causing LVADI. The frequency of LVADI in our cohort is higher than what has previously been reported, possibly due to higher vigilance. The MDROs MRSA, MDR *Pseudomonas aeruginosa*, and MDR Enterobacterales are prominent in this population. Further study is needed to elucidate the risk factors for acquiring MDRO LVADIs and contributing factors to mortality in LVAD pts.

**Disclosures:**

**All Authors**: No reported disclosures

